# Scrotal Trauma in a Hemophiliac: A Noisy Picture With a Minimalist Treatment

**DOI:** 10.7759/cureus.45975

**Published:** 2023-09-26

**Authors:** Jihad Lakssir, Yassine Gounni, Ahmed Ibrahimi, Hachem El Sayegh, Yassine Nouini

**Affiliations:** 1 Urology A, Ibn Sina University Hospital, Rabat, MAR

**Keywords:** haemostasis disorder, factor viii, medical treatment, scrotal trauma, hemophilia-a

## Abstract

Post-traumatic hematoma is often associated with severe, extensive injuries. In patients with blood coagulation disorders, hematoma can occur even after innocuous injuries. Among these coagulation disorders is hemophilia, an X-linked recessive disorder characterized by hemorrhagic manifestations. The aim of this article is to report a case of massive scrotal hematoma following trauma in a 24-year-old hemophiliac with no signs of severity who benefited from medical treatment with infusion of coagulation factors with a good clinico-biological evolution.

## Introduction

Scrotal trauma, more often closed, accounts for around 1% of cases and affects mainly young subjects between 10 and 30 years old. Secondary to direct impact with a projection of the testicle against the pubic arch, it is characterized by a typical clinical presentation in the emergency department.

Hemophilia is an X-linked recessive disorder, characterized by hemorrhagic manifestations linked to coagulation disorders, mainly an anomaly in factor VIII. It affects around 1/10000 people, with a predominance of males.

Facing these combined clinical pictures, the attitude remains unicist, consisting of surgical exploration in the case of testicular rupture or hematocele, with management of the underlying pathology.

The aim of this article is to report a case of a large scrotal hematoma in a hemophiliac patient with medical management.

## Case presentation

**Figure 1:**
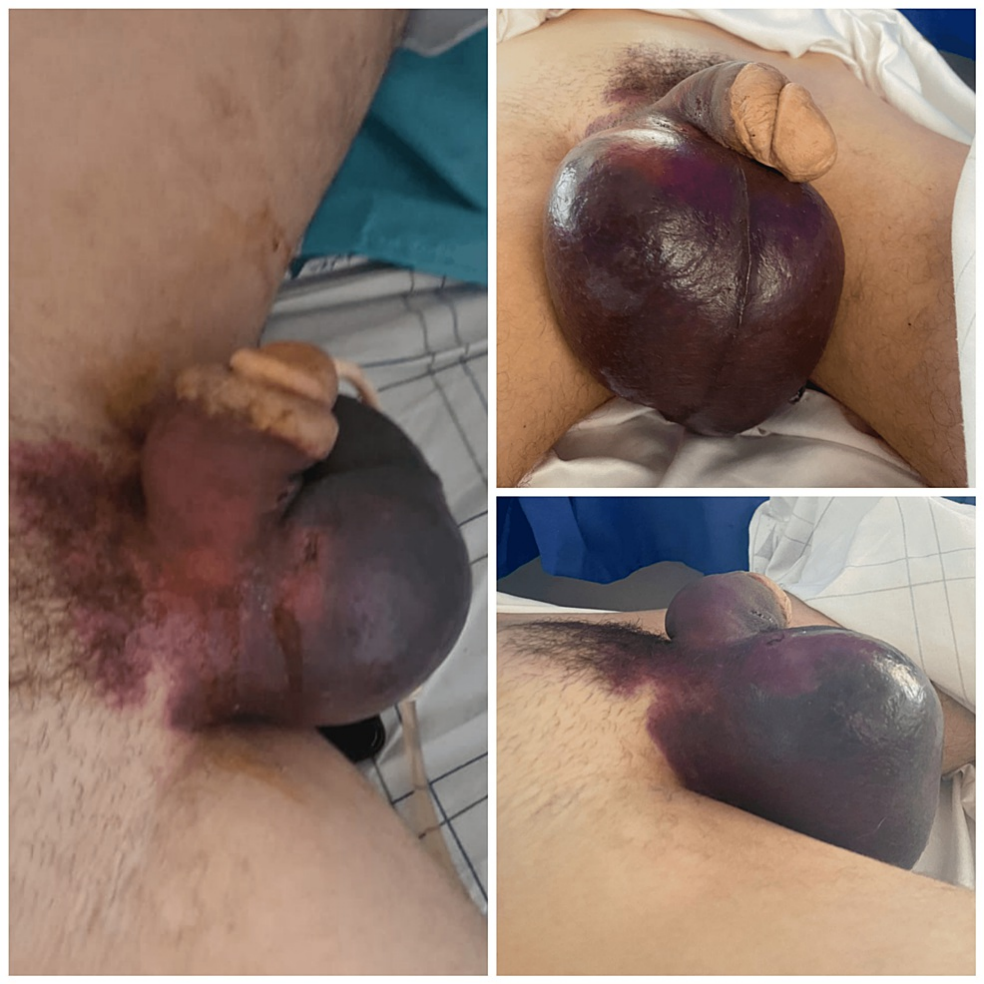
Image showing an ecchymotic large bilateral bursa with an extension to the inguinal region.

A 24-year-old patient, known as hemophiliac A, presented to the emergency department with scrotal trauma following minor trauma involving heavy lifting. General examination revealed a hemodynamically, respiratorily, and neurologically stable, and lower back swelling, while scrotal examination revealed a large bilateral bursa, ecchymotic in appearance, painful to palpation and extending to the right inguinal region (Figure 1).

**Figure 2:**
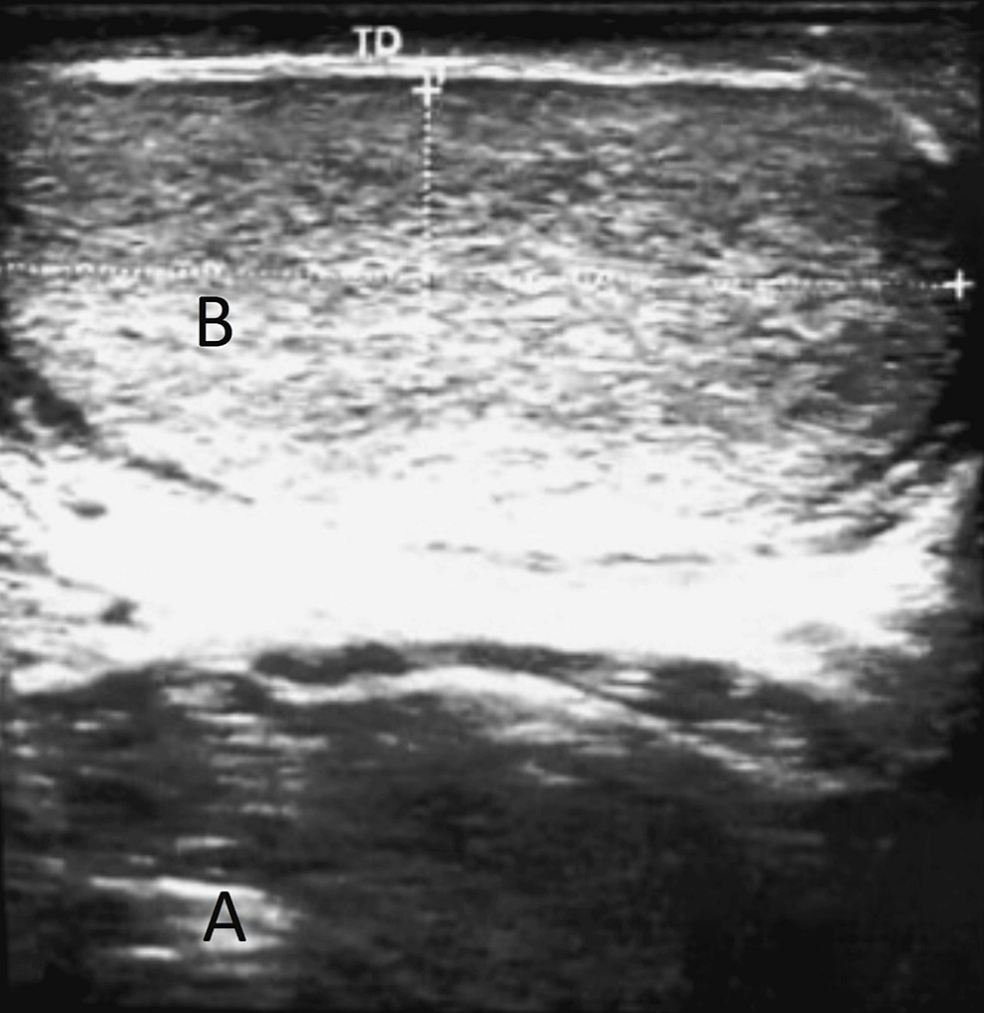
Image showing a large hematic collection of the scrotal envelopes (A) surrounding a normal-sized testicle (B).

A scrotal ultrasound, performed in the emergency department, revealed a large hematic collection of the scrotal envelopes surrounding the testes, fusing upwards at the level of the spermatic cords, with respect to the albuginea, and a normal-sized testicle, with regular contours and good vascularization (Figure 2).

Biologically, the hemoglobin was 8.2 g/dl, and the platelet count was 252.000 G/l, while the rest was with no particularity.

The patient was admitted to a hematology department specializing in hemophilia where clinical monitoring was instituted, and a medical treatment protocol was as follows: Octanate 1500 IU twice a day and Dynastat (Parecoxib) 40 mg twice a day, both for seven days.

**Figure 3:**
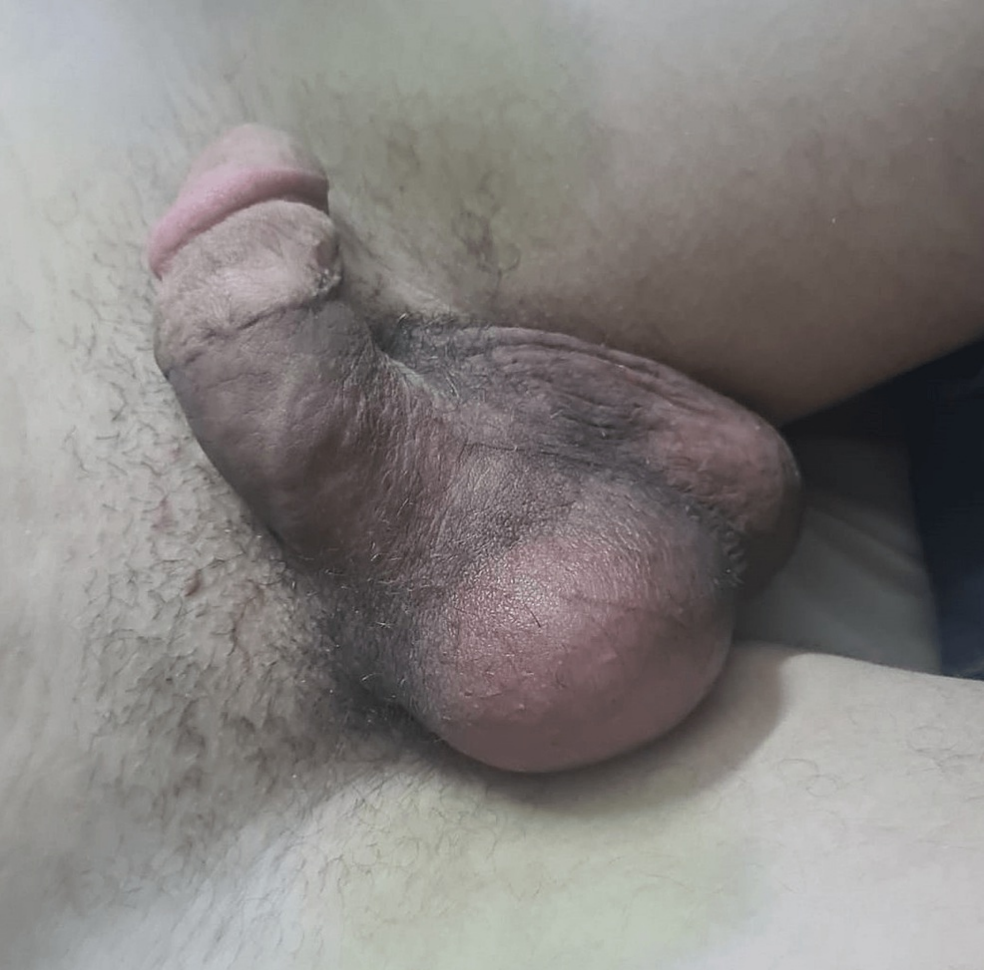
Image showing a complete regression of the ecchymotic appearance of the bursa.

Our attitude was conservative, with close monitoring of the patient, characterized by stabilization of the clinical picture during the first week and subsequent regression. A clinical and ultrasound reevaluation at one month showed complete normalization of the clinical appearance of the bursae (Figure 3).

## Discussion

Hemophilia is an X-linked hereditary disorder causing a coagulation disorder and is therefore responsible for bleeding that may be spontaneous or secondary to even minimal trauma [1].

To understand this pathology, we first need to understand the mechanism of hemostasis: when a blood vessel is breached, tissue factor meets circulating factor VII, initiating a coagulation cascade that leads to the formation of thrombin. Thrombin is an essential component of this coagulation process, which includes platelet activation and aggregation, enabling the collagen-exposed sub-endothelial surface to be sealed, and the formation of fibrin, which is subsequently used to stabilize the thrombus [2].

In addition, on the phospholipid membrane of these platelets, a coagulation cascade takes place in which Factor VIII and Factor IX are found, enabling the amplification and further propagation of thrombin production, thus triggering a 'Thrombin Burst' [2]. A defect in one of these factors is responsible for hemophilia.

Hemophilia is a genetic disorder, where type A is predominating in 80% of cases, in which the defective gene is found in the distal end of the long arm of the X chromosome [2]. The diagnosis of this pathology is evoked by a history of abnormal bleeding, including a positive family history, or by the chance discovery of an abnormal coagulation profile. Confirmation is based on the measurement of the residual coagulant activity of the factor in question.

This pathology is responsible for a noisy clinical picture, yet the trauma involved may be minimal, as observed in our context.

Management in this particular context is often complicated by this hemostasis disorder, requiring a multidisciplinary approach. However, it remains standardized in the case of closed scrotal trauma, where radiological exploration using scrotal ultrasound plays an important role in ruling out a hematocele or ruptured albuginea, with a reliability rate of 94% [3]. However, in the event of diagnostic doubt, surgical exploration becomes indispensable [4], enabling an early exploration to reduce the risk of orchiectomy and long-term complications such as testicular atrophy, testicular pain, and fertility problems.

Effective management of the underlying pathology is essential, based on the replacement of coagulation factors to achieve adequate levels to prevent or stop unnecessary bleeding.

As in our context, although well-managed prophylaxis reduces the risk of bleeding, it does not eliminate it. The World Federation of Hemophilia (WFH) has published recommendations (Table 1) for site-specific bleeding treatment [5]. However, this treatment must be adapted to obtain optimal individual results, which will depend on the site, the extent of bleeding, and the individual response.

**Table 1:** Common sites of bleeding in hemophilia and WFH recommended FVIII replacement therapy [5]

Site of bleeding	Typical target FVIII range (IU/dl)	Typical duration of replacement therapy (days since bleed or surgery)
Joint (60% of bleeding episodes)	40-60	1-2
Muscle (30% of bleeding episodes)	40-60	2-3
Gastrointestinal tract	80-100 (initial)	7-14
	50 (maintenance)	
Iliopsoas	80-100 (initial)	1-2
	30-60 (maintenance)	3-5
Throat and neck	80-100 (initial)	1-7
	50 (maintenance)	8-14
Renal	50	3-5
Central nervous system	80-100 (initial)	1-7
	50 (maintenance)	8-21
Deep laceration	50	5-7
Surgery (major)	50-80 (pre-op)	Pre-op
	60-80 (post-op)	1-3
	40-60 (post-op)	4-6
	30-50 (post-op)	7-14
Surgery (minor)	50-80 (pre-op)	Pre-op
	30-80 (post-op)	1-5

Thus, hemophiliac patients with closed scrotal trauma do not automatically require surgical treatment when the clinico-radiological data are reassuring.

## Conclusions

Bleeding and hematoma formation following trauma are common in hemophilia patients, for whom the administration of factor VIII is recommended.

In the absence of serious signs, medical treatment with regular monitoring is justified. However, in the event of clinical or ultrasound doubt, surgical exploration should be conducted as soon as possible.
